# Turkish and Moroccan Dutch women’s views of using a self-sampling kit for human papillomavirus testing as a tool for cervical cancer screening: What are the barriers and the motivators?

**DOI:** 10.1177/17455065211065873

**Published:** 2021-12-13

**Authors:** Femke Hilverda, Katleen Fissers, Thijs van den Broek

**Affiliations:** Department of Socio-Medical Sciences, Erasmus School of Health Policy & Management, Erasmus University Rotterdam, Rotterdam, The Netherlands

**Keywords:** cervical cancer screening, HPV, self-sampling, theory of planned behavior, Turkish and Moroccan Dutch women

## Abstract

**Objective:**

This study explores barriers and motivators to use self-sampling kits for human papillomavirus testing for cervical cancer screening as perceived by Dutch women of Turkish and Moroccan origin living in the Netherlands.

**Methods:**

A total of 11 in-depth semi-structured interviews were conducted and structured according to the theory of planned behavior.

**Results:**

Findings suggest that self-sampling may lift important barriers hampering traditional cervical cancer screening, such as those related to shame and chastity. However, self-sampling raises its own barriers too. Most importantly, some women fear that self-sampling may harm virginity. Some women also do not feel confident about their ability to properly use the self-sampling kit, but fears about the inability to properly use it often fade away upon having seen the self-sampling kit. Moreover, results show that knowledge about cervical cancer and its origin is limited, which may undermine women’s willingness to participate in a screening program.

**Conclusions:**

These results suggest that communication strategies to encourage using self-sampling kits among women of Turkish and Moroccan origin could benefit from culturally sensitive approaches, for example, by placing emphasis on issues such as virginity and chastity. Consistent with a recent advice of the Health Council of the Netherlands, the kit could furthermore be sent to eligible women as a standard procedure, rather than upon request. This could reduce hassle and doubts about women’s ability to use the self-sampling kit. Finally, educating women about the importance of screening to prevent cervical cancer is needed to foster informed decision-making.

## Introduction

The development of cervical cancer is a slow process caused by an infection with high-risk human papillomavirus (HPV) through sexual intercourse. Symptoms of cervical cancer are often only manifested in an advanced stage of the disease. For this reason, early detection is of utmost importance. Screening programs to detect cervical precancer (i.e. changes to the cervical cells that might develop into cancer) reduce mortality and incidence of cervical cancer.^1,2^ The Netherlands implemented a nationwide uniform screening program in 1996. When a woman turns 30 years old, she gets an invitation letter in which she is asked to make an appointment for screening, regardless of whether she is sexually active. After that, screening invitations are sent out every 5 years until a woman reaches the age of 60.^3^

In the Netherlands, the 1-year incidence of cervical cancer was 9.0 per 100,000 women in 2017. The mortality rate of cervical cancer in the Netherlands varies between 2.3 and 2.6 per 100,000 women per year.^3^ It is estimated that without the screening program, the mortality rate would be more than twice as high, and up to 500 women would die on a yearly basis.^3^ This shows that screening is effective in limiting mortality of cervical cancer.^4,5^

Unfortunately, not all eligible women participate in the screening program and about half of the women with cervical cancer diagnosis did not participate.^6^ Participation rates are particularly low in women with a low socio-economic status and women with a non-Western migration background.^7,8^ The low screening participation rates are worrying, because research shows that the cervical cancer incidence is higher for women with a non-Western migration background than for native Dutch women.^9^

Together with women of Surinamese origin, women of Turkish and Moroccan origins are the largest groups of women with a non-Western migration background in the Netherlands. The estimated rates of participation in the screening program are approximately 64% among Turkish Dutch women and 53% among Moroccan Dutch women, which is considerably lower than rates reported for native Dutch women and for women of Surinamese, Antillean, and Aruban origin.^10,11^

The low use of screening uptake in the former groups has been attributed to a range of factors. These include a lack of knowledge and awareness of cervical cancer,^12–14^ poor command of the Dutch language,^12,13^ reluctance to visit a male general practitioner, fatalism, the association of cervical cancer with lack of femininity and infertility,^14^ and emotional responses toward screening, such as shame and worry.^15,16^ Positive social norms and social network, on the other hand, were found to be important facilitators of screening behavior.^14,16,17^

It has been suggested that self-sampling kits testing might provide new opportunities to encourage participation in cervical cancer screening among groups of women with lagging participation rates.^5,18–22^ A self-sampling kit screens for HPV. If the test is positive, which, overall, occurs in approximately 10% of the cases,^3^ a follow-up traditional smear test needs to be performed.^5^ When women are able to swab themselves as an alternative to visiting a healthcare professional, multiple barriers experienced, such as having a male general practitioner and the experience of shame, may be lifted.^12–14^ Given that women with non-Western migration background typically experience such barriers relatively strongly, self-sampling could potentially be particularly relevant for this group of women.

Since 2017, the option to use a self-sampling kit is made available in the Dutch national screening program. Women can request to receive a self-sampling kit online (website), via email or by phone. Initially, this option was only presented in the reminder sent out to women who did not respond to the initial invitation. Since recently, the option of self-sampling is already introduced in the initial invitation that women receive.^23^

However, little is known about how women with a Turkish or Moroccan background living in the Netherlands think about self-sampling tests for HPV.^14^ The current study addresses this knowledge gap by exploring the motivators and barriers of self-sampling kits for HPV testing as perceived by Turkish and Moroccan women living in the Netherlands. A recent study of Hamdiui et al.^14^ is, to our best knowledge, the only study focusing on the willingness of women with a non-Western migration background to partake in the Dutch cervical cancer screening program taking self-sampling for HPV into account. The authors found that women were uncertain about whether they would be able to self-sample correctly and preferred a professionally taken sample. While Hamdiui et al. did explore attitudes toward self-sampling, it was not the central theme in their study, unlike in the current study. Moreover, the authors used focus groups as their mode of data collection. Compared to individual interviews, focus groups offer unique opportunities to gain valuable insights into attitudes regarding the topic of interest, most notably because the interaction between focus group members may trigger them to provide richer information.^24^ However, the method also comes with its own drawbacks. Importantly, group processes may hamper respondents’ willingness to express their individual views when these views are not consistent with the dominant view in the focus group.^25^ Also, the time for an individual respondent in a focus group to share information is much more limited than in a one-on-one session.^26^ It is therefore not surprising that empirical comparisons of focus groups and individual interviews typically show that, per respondent, more unique information is gained from individual interviews than from focus groups.^27–29^ The current study in which individual interviews are conducted with HPV self-sampling as the central theme is therefore likely to yield insights that are complementary to those gained by Hamdiui et al.^14^ and to deepen the understanding of the barriers and motivators of self-sampling.

In our study, the motivators and barriers to use self-sampling kits for HPV testing as perceived by Turkish and Moroccan women living in the Netherlands are examined to enable us to make culturally sensitive recommendations aimed at encouraging screening among women with a migration background.^30^ Insights gained may guide the adaptation of information provision about the screening program to increase informed decision-making and screening participation, and, ultimately, to reduce cervical cancer mortality. In addition, practical recommendations on how communication strategies might contribute to higher cervical cancer screening rates will be discussed.

## Materials and methods

### Participants

**Figure 1.**
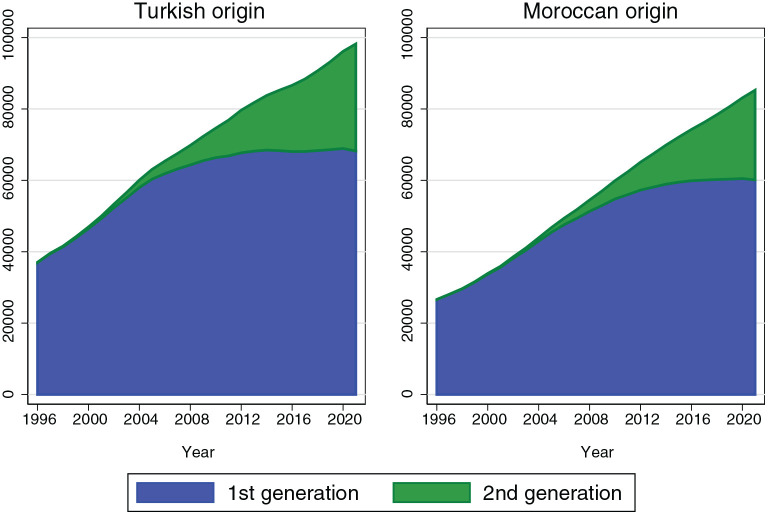
Turkish and Moroccan origin women aged 30–60 years in the Netherlands.

The target group for the interviews consisted of women of Turkish and Moroccan origin living in the Netherlands. Recruitment took place via a midwifery practice in Zaandam, the Netherlands. A total of 20 women were purposively approached face-to-face or by phone by one of the midwives working at this practice and informed about the opportunity to participate in an interview study about cervical cancer screening and self-sampling. Women who were interested (*n* = 11) received an information letter via email. These women were (ex)-clients of the practice or collaboration partners. Women who declined participation did so because they did not have time (e.g. they were busy with the newborn), thought the subject was difficult to talk about, or were not interested. By means of snowballing one additional woman, who did not have children, was included. Unfortunately, the COVID-19 outbreak precluded inclusion of more women without children. Women were included in this study if they were from Turkish or Moroccan origin, and aged between 20 and 60 years old. Both first- (women born in Turkey or Morocco) and second-generation (women with at least one parent born in Turkey or Morocco) migrants were included. This was because the proportion of second-generation migrants aged between 30 and 60 years has been growing rapidly over the last years as depicted in Figure 1. In addition, second-generation migrants might act as advisor for first-generation migrants in connecting them with the screening program.^31^ The age range was chosen to include the perceptions of women age-eligible for cervical cancer screening as well as women who will be invited soon (within 10 years from now). Women who were aged above 60 were excluded, as screening ends at this age.

Single time interviews were scheduled based on the availability of the women. Face-to-face interviews took place at a location chosen by the interviewee, most often the interviewee’s home. One woman preferred to be interviewed by phone. A total of 12 women participated. Two women were interviewed together (mother and daughter), and the other interviews were one-on-one interviews. The duration of the interviews was on average 45 min.

### Data collection and analysis

In-depth qualitative semi-structured interviews were used as a method of data collection. All participants were informed about the purpose of this study verbally by phone beforehand and verbally as well as written at the start of the interviews. It was emphasized that data would be anonymized and that participants could withdraw from participation at any time without (negative) consequences. Verbal and written informed consent was obtained to record the interview and to use the data gathered in the interview before starting the interview recording. During the interviews, the focus was on the conversation, and therefore, the interviewer did not make extensive notes, but rather recorded the interviews and focused on having natural conversation with the interviewee. The female interviewer (K.F.), one of the midwives of the practice (BSc degree), was familiar with the women who participated, except the woman who was recruited indirectly via snowballing. At the time of data collection, the interviewer was attending a master program in which she received training on conducting qualitative research.

Interviews were held using a semi-structured format. The interview guide, though not pilot tested, was extensively discussed in the research team before data collection. First, women’s pre-existing knowledge on cervical cancer screening was probed. A brief explanation was given about the screening program and self-sampling. The self-sampling kit was shown and women were invited to examine the kit more closely and hold it. The interviewer used responses of the interviewees to guide more in-depth questioning during the interviews.

The interviews were structured around the main determinants of the theory of planned behavior (TPB).^32^ TPB states that behavior is predicted by behavioral intention, which, in turn, is determined by an individual attitude, social norms, and perceived behavioral control. Attitude refers to an individual’s general opinion on a behavioral action in terms of favorability (i.e. how positive or negative is the behavior appraised by an individual?). Social norms describe the influences of the social environment, such as the social pressure someone feels to engage in a particular behavior or the individual’s perception of what others do. Perceived behavioral control explains the individual’s beliefs in the capability to perform a certain action or behavior.

In addition, knowledge was added as a central theme that was discussed, because research shows that lack of awareness and poor knowledge about cancer screening are important reasons why women do not attend the cervical cancer screening program.^14,33^ The TPB was found useful to explain cervical cancer screening participation in previous research^34,35^ and therefore chosen as a theoretical framework for current purpose.

Data saturation occurred after approximately eight to nine interviews. Interviewing continued until 12 women participated to ensure that no new information was missed. Women received a small present (flowers) for their participation. Women were offered the opportunity to look into their transcripts and receive a copy of the results of the study. The results were sent to nine women. No additional feedback was provided.

The interviews were audio recorded and transcribed verbatim. Subsequently, one researcher (K.F.) performed a thematic content analysis^36^ of the data by means of Atlas.ti. Themes and codes were frequently discussed with and checked by another researcher (F.H.) to increase the reliability of the analysis. First, interviews were open coded. In this phase, codes were inductively based on the answers respondents provided. Then, these codes were deductively grouped into themes. The concepts of the TPB and the additional concept “knowledge” were used to structure the themes according to these determinants of behavior. Quotes used to illustrate themes were translated from Dutch by researcher F.H.

### Ethics statement

The Research Ethics Review Committee of the Erasmus School of Health Policy and Management confirmed that the current study did not require approval from a Medical Ethics Review Committee (Reference: ETH2122-021). The study did not fall within the scope of the Dutch WMO law (Medical Research Involving Human Participants Law) because it did not involve manipulation or medical data of patients. All procedures performed were in accordance with the ethical standards of the institution (EUR) and in line with the Helsinki declaration on ethical standards. Women participated on a voluntary basis and were able to withdraw at any time without (negative) consequences. Prior to the interview, written and verbal informed consent was obtained. During data analysis, names and privacy-sensitive information were removed to ensure that data could not be traced back to the participants.

## Results

Interviews were conducted with seven women of Turkish origin and five women of Moroccan origin. All women, except one, were second-generation immigrants and all spoke Dutch fluently. Eight women were within the target group of the Dutch cancer screening program, that is, aged 30 years or older. Most of the women were married and had children. An overview of the characteristics of the respondents is presented in Table 1.

**Table 1.** Participant characteristics.

Participant	Age	Ethnicity	Marital status	Number of children	Participation in cervical cancer screening program	Notes
1	33	Turkish	Married	3	Yes	
2	53	Turkish	Divorced	2	Yes	Healthcare professional; first generation
3	21	Turkish	Single	0	No, not yet invited	
4	38	Turkish	Married	3	Yes	Not religious
5	29	Turkish	Married	2	No, not yet invited	
6	29	Turkish	Married	1	Tested outside the program, because experiencing complaints	Healthcare professional; currently pregnant
7	32	Turkish	Married	2	No, invited but pregnant at that moment	Healthcare professional
8	43	Moroccan	Married	4	Yes	Used self-sampling kit
9	34	Moroccan	Married	3	No, invited but pregnant at that moment.	
10	26	Moroccan	Married	2	No, not yet invited	
11	30	Moroccan	Married	2	Tested outside the program, because experiencing complaints	Currently pregnant
12	39	Moroccan	Married	3	No, decided not to participate	

The thematic analysis identified experienced barriers and motivators regarding the use a self-sampling kit to test for HPV as tool for cervical cancer screening. In the following section, these barriers and motivators are elaborated upon from a TPB perspective, whereby the additional element of knowledge in addition to the TPB concepts is considered as a major theme. An overview of the results can be found in Table 2, showing the barriers and motivators per determinant.

**Table 2.** Overview of the results.

Determinants	Barriers	Motivators
Knowledge	• Little knowledge about cervical cancer • Self-sampling is unknown	• Information-seeking capability • More information in own language will possibly stimulate women without a good command of the Dutch language to search for information
Attitude	• Concerns about validity • Stressful waiting time between self-sampling and cytology	• Lifts barriers related to screening: pain, embarrassment, and chastity problems
Social norms	• Virginity issues: (1) Self-sampling might affect virginity and (2) may be associated with sexual activity before marriage • HPV-positive test result implies sexual activity of one or both partners outside marriage	• Discuss with other women or partners • Religion stimulates to take care of your body • Information provision by female care professionals (with the same cultural background/religion) might motivate women to partake, especially information about virginity might stimulate participation of unmarried women
Perceived behavioral control	• Older women might experience difficulties because they lack knowledge of female anatomy • Receiving the self-sampling kit requires action, which might be a more prominent barrier for women without good command of the Dutch language	• Women feel capable after seeing the self-sampling kit and reading the manual • Sending the kit with the invitation possibly helps to overcome barriers related to capability and logistics

HPV: human papillomavirus.

### Beliefs about the cervical cancer screening program

Both the Turkish and Moroccan origin women were generally very positive about the Dutch screening program. The knowledge about the program was, however, limited, particularly among women who had not yet been invited for screening. Women obtained information about the program mainly through the invitation(s). Although the current study’s respondents—who were mostly second-generation migrants and spoke Dutch fluently—had limited knowledge about the program themselves, they suspected that there would be an even stronger lack of knowledge among first-generation migrants because of language barriers, low literacy, and limited digital skills. The taboo surrounding female sexuality was also named as a possible barrier for older migrants. Respondent 4 about this taboo: “My generation, this is a different time now, but the generation before me was taught not to talk about issues like that, about childbirth and things like that, you had to find that out yourself, discover it and go.”

Most of the women had little knowledge about cervical cancer and the cause hereof. Respondent 6, for example, told the interviewer that she did not know what causes cervical cancer. When later asked about transmission of HPV, she indicated again that she did not know how the transmission takes place. While knowledge was limited, several of the women indicated that participation in the screening program is important, like respondent 7: “I think it is very important, screening, for everyone, but I would also be eager to know if everything is alright.” Respondent 8 was even more firm with respect to participating in the screening program, and she stated, “I wouldn’t understand how someone my age would ignore that. That doesn’t get through to me. I mean it’s such an important thing.”

However, the intention to participate not always resulted in actual participation, for example, due to a pregnancy at the time of invitation or practical reasons, such as difficulties with transport or with finding a babysitter. Moreover, a small number of women indicated that they would consider their risk of HPV to decide whether to participate. For example, respondent 3 explained that participation depends on whether she is still single and living with her mother or whether she is married: “Suppose I am in the same situation as now, I think it [cervical cancer screening] is redundant (. . .). If I am married on my 30th, then yes, of course.” Also, respondent 12 explained that she decided not to participate because her risk of HPV is low considering she is involved in a loyal marriage:

It concerns people who have different sexual relationships. That is not the case in my world, anyway. I am married and I assume that my husband is faithful to me. From what I understand, that [having sex] is actually the way you can get the virus.

### Knowledge of self-sampling

The option of self-sampling was unknown to almost all respondents. These respondents explained that they had not heard about this possibility before they were contacted for an interview on this topic. Only one woman was familiar with self-sampling. This woman used a self-sampling kit, but did not remember applying for it. The test may have been sent to her as part of a pilot study.

One aspect of self-sampling that the women had little knowledge about was the link between HPV and cervical cancer. Most women did not realize that the self-sampling kit only tests for HPV and that a smear test is needed if the result is HPV-positive. Waiting time between an HPV-positive result and the follow-up cytology may cause concern. However, when the interviewer explained this, most women argued that they estimated that their risk to be HPV-positive was low and that they therefore did not consider the waiting time for a potential follow-up cytology to be a relevant factor for them.

Although women in this study had little knowledge about self-sampling, they indicated that they could easily search for information if they needed to. They had a good command of the Dutch language and good health literacy. They explained that searching information might be difficult for women who experience a language barrier. The current information supplied—a brief reference to the website in the invitation in Turkish and Moroccan language—did not trigger respondents to search for information themselves, because the urgency of testing and severity of the disease was not emphasized strongly enough in the information received.

### Attitude

Several women indicated that they would accept having a smear test if no alternative option would be available, but that they would prefer self-sampling. Attitudes toward the option of self-sampling to test for HPV were typically favorable. The main reason for this was that some women dreaded having a smear test due to a previous unpleasant experience, such as respondent 12: “The only thing I ever did was, my God, thinking about it makes me warm, (. . .) I was examined internally and then a speculum was used. Yes, that is a bad experience, I really perceive that as something terrible . . . .” Several women believed that the self-sampling kit offers a solution for the barriers they experienced with the smear test: pain, embarrassment, and concerns about chastity.

First, some women indicated that they believed that self-sampling was painless and less unpleasant than a smear test or internal examination. Respondent 8 explained that she felt less pain and discomfort when she had control over the action herself:

That speculum, I just think it is terrible, I tell you honestly (. . .) that way of insertion, that stretching, while if you use such a stick [self-sampling kit], because it is narrow . . . you can already feel how deep you can go with such a brush and I found that much more pleasant than with a speculum that tears everything open so to say.

Second, about half of the women preferred self-sampling over a smear test because it takes away the barrier of shame toward healthcare practitioners. Respondent 1 told,

Yes of course, not pleasant, it is . . . yes maybe indeed the Turkish culture a bit. It is not pleasant to be so open, even if it is your doctor. I don’t really know her (. . .) I almost never go to my doctor and then you suddenly have to do such a test. That didn’t feel right of course.

Several women indicated that the option to take the sample in the privacy of their own home offers a solution to issues related to embarrassment. Respondent 3 explained, “Yes definitely [I would use the self-sample test], it doesn’t look difficult, I would prefer taking the sample myself over going to the GP, I’d feel a bit embarrassed to go to the GP.”

Third, some women thought that self-sampling provides a solution to chastity concerns. Chastity involves more than just virginity, which can be defined as not yet having had sexual interaction. Virginity is considered important in Muslim societies, such as Morocco and Turkey. While the definition of virginity is straightforward, the symbolic value of virginity is more complex and related to the broader concept of chastity of women. Chastity also involves ideas about the appropriate behavior of virgins, such as not having contact or friendships with men outside their family and not showing an interest in men sexually.^37^ In this way, some respondents felt that it is their duty to require a female doctor whenever possible, while others indicated that according to the Quran there are no issues regarding chastity in relation to consultation a healthcare professional. Respondent 8 explained how self-sampling kits are a good solution to problems related to chastity. She believed self-sampling to be halal (i.e. pure and allowed by the Quran) mainly because you do not have get in touch with anyone and it is not needed to expose your body to a doctor (or anyone else):

If you get such a self-sampling kit at home, you don’t even have to go to a doctor, so it is completely halal. It’s just private, you do it at home, no one is there except your partner or an acquaintance who helps you, if you don’t know exactly how or what to do. So this is the best solution for me.

While women were positive about self-sampling as it lifted important barriers associated with smear tests, some women doubted the validity and reliability of self-sampling. They needed to be convinced that the quality of the sample taken themselves is the same as when a professional takes the sample. Respondent 9 told, “I want to be sure that the sample is taken correctly and that the results are reliable.” Two women indicated that the self-sampling kit looked so easy that they doubted its reliability. Respondent 2, a Turkish woman, explained,

I think good information is very important. I think if I get something like this self-sampling kit in the mailbox without information I would think “Is this trustworthy?” Come on, I don’t believe in it, this looks like some kind of toy, something like that.

### Social norms

Most women in this study valued consultation of important others before deciding whether or not to use a self-sampling kit. They felt able to talk easily with other women and with their partners about this topic. The opinion of others close to them provided important guidance for several respondents, for instance, for respondent 3: “Yes I am such a person, first ask my environment why would you do that or not? Yes, so I think my social environment plays a major role for me.”

Also the broader social environment, in this case, their Islamic religious background, was important in shaping women’s behavioral intention to self-sample for HPV. One respondent emphasized that according to Islam individuals are expected to live a healthy life: “It is our duty to keep our body healthy” (respondent 12). Self-sampling fits with this notion. It is important to note, however, that although several women consulted others and took into account what their religion prescribed, most women emphasized that, ultimately, the decision on whether or not to participate in the screening programs was theirs to make.

A major barrier related to using a self-sampling kit were concerns about virginity. Several women highlighted that according to Islam, it is important that men and women do not have sexual intercourse before marriage. The issue with virginity was two-sided. First, some women were concerned that self-sampling could affect their virginity. Second, having had sexual relationships may be inferred from unmarried women’s participation in self-sampling. With regard to the former, women had different ideas about self-sampling affecting virginity. For example, respondent 8 explained how women who are still virgins might be anxious to self-sample, because they may believe that self-sampling affects virginity:

“You will feel that fear of ‘oh dear and what about my virginity,” doing such a test, probably not . . . The young generation will hear repeatedly from childhood onwards “be careful, no one can touch it [your vagina], you can’t touch it [your vagina] because nobody will accept you anymore, you will never get married.” That’s a kind of trauma.

However, several women stated that while devout Muslims place extreme value on maintaining virginity until marriage, Muslims who are less strict experience more room for maneuver. The norms that women impose on themselves or feel imposed by their social environment strongly depend on their own interpretation of the Quran. Respondent 12 explained that there is always room for own conclusions within religion: “You know, on a religious level, especially with these kinds of things, there is always room to draw your own conclusions (. . .) And in this case the most important thing is to know that health is very important.” Similarly, respondent 6 told that her health is more important than virginity. She says, “My health comes first and I don’t have to justify myself for anything [i.e. not being a virgin], because I believe in my God (. . .) That it is between me and my God.”

In addition to self-sampling potentially harming virginity, some women thought that sexual activity could be inferred from performing a self-sampling. This concern was mentioned in response to the interviewer’s explanation that HPV is transmitted via sexual contact. According to these women, since HPV is only transmitted via sexual intercourse, wanting to perform a self-test for HPV only made sense if a woman is sexually active. Some women noted that unmarried women who want to use a self-sampling kit and still live at their parents may face a dilemma because the self-sampling kit is sent to their house. This is because it may be inferred from receipt of the self-sampling kit that a woman is sexually active before marriage. Respondent 3 explained how it would be shameful if people find out women are sexually active before marriage:

Some women over 30 who still live at home . . . who are a bit ashamed of this. That could well play a role, if a letter or bill is sent to their house and then the other housemates may find out after all.

Moreover, some women believed that participation could lead to issues of distrust within a marriage. After all, having an HPV-positive result, or even the mere participation in the screening program, may suggest that either spouse has had sexual relations before or outside of marriage. Most respondents had not thought about this yet, but when asked said that the fear of being confronted with an HPV-positive result would not withhold them from self-sampling.

### Perceived behavioral control

When looking at the demo model, most women immediately felt confident that they were capable to do this correctly. Some women initially had doubts, but after reading the manual, they were convinced too. For example, while respondent 9 initially said, “Do you know what I think about such a self-sampling kit? Wait, maybe I will not be doing it correctly or something. And then the result is not reliable. A fear that I have (. . .),” after studying the test and manual she concluded, “But it looks easy . . . yes ok, it’s simple as that!.” Most women told that they only realized how simple taking the sample is when they had the self-sampling kit in their hands, which was something they had not expected in advance. Therefore, they expressed a preference for receiving the self-sample kit directly along with the invitation. Respondent 9 said about this:

No . . . no, they really should send it right away so that you get a clear picture indeed, otherwise I would still have made an appointment with the doctor. (. . .) If you see the self-sampling kit then you think “Ok, I can do this.”

Although most women expressed feeling confident about using the self-sampling kit, this was not the case for all women. Respondent 5 believed that though she would probably opt for self-sampling, she would possibly still doubt whether she did it correctly: “When I go to the doctor, I have certainty, but when I do it myself, I am like ‘Ok, I did it, but did I do it correctly?’” Moreover, women who had experience as a healthcare professional believed that for some, especially older, women taking a self-sample would be difficult, because they have little knowledge about female anatomy. Respondent 7 explained, “I think they would rather go to the doctor than mess around themselves, because they are afraid (. . .) I think it certainly plays a role that they just don’t know what they look like down there.” The women who did not work as a healthcare professional did not mention this barrier.

In addition to perceived capability, logistics played a role in perceived behavioral control. The fact that women actively need to apply for a self-sampling kit was perceived as a barrier by some women. Respondent 11 said about this:

If the self-sampling kit was included in the invitation, I would be more likely to do it than if I have to apply for it first (. . .) While if you already have the self-sampling kit right away, you are be able to do it right away . . . .

Some women thought that this barrier is even more prominent for women who do not have sufficient command of the Dutch language or low (health) literacy.

#### Intention to self-sample

The expressed intention to self-sample was high: ten women preferred to use the self-sampling kit, one woman was still uncertain whether to participate by means of a smear test or by self-sampling mainly, because she doubted that she could do the test correctly. One woman indicated that she would refrain from participating in the screening program altogether, because she considered herself as not at great risk of being HPV infected. Women were asked to think about ways to encourage other women of Turkish and Moroccan origin to participate in the screening program by using a self-sampling kit. Three main recommendations were discussed.

First, women believed that tailored information provision could increase the intention to participate. This included more explicit communication of the consequences of late detection of cervical cancer. Women thought that communicating the risks of HPV and the benefits of screening encourages participation. Reading testimonials and experiences of other women with HPV-positive test results may encourage participation. For women with a language barrier, it seemed useful to include more text in their own language in the invitation letter, so that, they are motivated to search for information online themselves.

Second, women indicated that unmarried women might be convinced if a prominent person, preferably someone with the same religious background, explains that self-sampling does not pose a threat to virginity. Care professionals may play an important role in this. Respondents 2, 6, and 7, who were care professionals in maternity care explained that they were often approached by other women with a migration background with questions regarding sexuality and the female body, also outside their work, and noticed that they were considered role models. Respondent 2 told, “They [women with a migration background] ask me a lot in that area, they think I know a lot about it, so they ask me a lot about that kind of things.”

Third, women expected that the intention to participate may be increased if the self-sampling kit is sent to women as standard procedure. By sending the self-sampling kit by default the barrier posed by having to apply for it is overcome.

## Discussion

Early detection is very important to prevent development of cervical cancer^2^ and to decrease mortality and incidence of this type of cancer.^1^ However, not all eligible women participate in the screening program,^6^ particularly women with a low socio-economic status and women with a non-Western migration background.^7,14,38^ The self-sampling kit for HPV testing might offer a solution for this problem. In this study, we therefore explored the perceived barriers and motivators to use of self-sampling kits for HPV testing as tool for cervical cancer screening as perceived by Turkish and Moroccan Dutch women living in the Netherlands. A total of 11 interviews with 12 women were conducted and analyzed using the TPB as theoretical framework.

Our findings suggest that self-sampling for HPV offers a unique opportunity to encourage Turkish and Moroccan Dutch women to partake in the cervical cancer screening program. Several aspects of self-sampling were highlighted as instrumental in motivating women to participate in the screening program.

Most importantly, many women perceived that self-sampling allowed them to circumvent barriers hampering participation that were associated with smear tests, such as pain, embarrassment,^15,16^ and chastity concerns.^14,39,40^ Moreover, information provision by female healthcare professionals (preferably with the same cultural background/religion) might motivate Turkish and Moroccan Dutch women living in the Netherlands to partake. More specifically, the interviewed women highlighted that they greatly valued the opinions of care professionals, and information about the (absence of) implications of self-sampling for virginity provided by care professionals might reduce important concerns present particularly among unmarried women. In addition, the partner and other women in the social environment provide avenues through which women may be encouraged to partake, as does a religion-based appeal that emphasizes the importance of taking good care of one’s body, which is in line with previous research on screening participation in general^13,16,17^ and cervical cancer screening among Turkish and Moroccan Dutch women, in particular.^14^

However, women in this study also pointed out that self-sampling for HPV testing came with its own barriers hampering participation. It is important to consider these barriers to implement self-sampling successfully. Barriers included the idea that self-sampling may harm virginity. In addition, women believed that self-sampling may be associated with sexual activity before marriage. It is, however, uncertain how relevant this perception is, because women did often not know that HPV is transmitted via sexual contact. Second, women doubted whether they were capable of using the self-sampling kit properly. Other research^14,41^ also found that, although women from Turkish and Moroccan backgrounds believed that self-sampling is easy and accessible, they doubted whether they could perform the self-sampling correctly. Our study showed that showing the self-sampling kit substantially reduced most women’s doubts. The Health Council of the Netherlands^42^ recently advised that the kit be sent to eligible women as a standard procedure along with the invitation, rather than only upon request. The findings presented here suggest that this could help to overcome the barriers related to perceived ability to use the kit and to logistics. Previous studies already suggested to implement the self-sampling kit as primary screening method.^43,44^ Finally, some women expressed concerns about the validity and reliability of the self-sampling kit to test for HPV. Nevertheless, women in this study preferred self-sampling over traditional cervical cancer screening, which suggests that the aforementioned perceived benefits of self-sampling outweigh the perceived barriers to screening that it raises.

A particularly interesting finding from the interviews was that women of Turkish and Moroccan origins expressed little knowledge about cervical cancer and the screening program, which is consistent with findings from previous research conducted among this group.^13,41,45^ Only one woman was familiar with the option of using self-sampling kit within the Dutch screening program. This might be explained by the fact that the self-sampling kit has only been offered since 3 years. Although women in our study tended to have little knowledge, most of them felt competent with regard to searching information. They also felt, however, that finding relevant information may be difficult for migrant women who experience a language barrier. Information provision in the language that these women feel most comfortable with may encourage women without a good command of the Dutch language to search for information. In this way, barriers related to lacking knowledge about HPV and cervical cancer may be reduced.^12–14^ Health communicators and healthcare professionals should explore culturally sensitive approaches to inform women about cervical cancer screening to foster informed decision-making. Attempts to encourage the use of self-sampling kits among women of Turkish and Moroccan origin could benefit from communication strategies that consider the cultural background of these women, for example, by placing emphasis on issues such as virginity and chastity. Moreover, showing the self-sampling kit, and thereby convincing the women that they are able to self-sample, may encourage participation.

To our best knowledge, this study is the first to provide in-depth insights into the motivators and barriers related to self-sampling for HPV testing as experienced by Turkish and Moroccan women in the Netherlands. In this way, our study is a valuable addition to the study of Hamdiui et al.^14^ that explored the opportunities of self-sampling, but mainly focused on reasons for participation in the cervical cancer program by means of a smear test. The added value of our study concerns not only the difference in focus, which led to more in-depth information regarding perceptions on self-sampling, but also involves the procedure of showing Turkish and Moroccan Dutch women the self-sample kit. One of our main findings is that doubts about performing the self-sampling correctly may be substantially reduced by simply showing the test or by sending it to eligible women by default rather than only upon request. Moreover, the importance of the social environment in encouraging self-sampling and the assisting role female care professionals could have in information provision extends the findings of Hamdiui et al.,^14^ who already highlighted the importance of social norms and social support in relation to traditional cervical cancer screening.

Nevertheless, some limitations of this study should be acknowledged. Most importantly, the composition of our sample should be considered. The women in our study were mainly second-generation migrants who were highly educated and had a good commend of the Dutch language. Perceived barriers and motivators might be different for women who experience a language barrier or are lower educated. In addition, this study focuses on intentions rather than behavior. Future research is needed to examine whether women actually translate their intention into behavior.

## Conclusion

Self-sampling for HPV might be helpful to increase the participation rate of the Dutch cervical cancer screening program among women of Turkish and Moroccan origins living in the Netherlands. However, although self-sampling kits for HPV testing lifts important barriers related to traditional cervical cancer screening, self-sampling raises barriers too. Health communicators and healthcare professionals are challenged to explore culturally sensitive approaches to encourage screening participation using self-sampling kits, for example, by addressing concerns about issues such as virginity and chastity. Raising the level of knowledge about cervical cancer (screening) among women of Turkish and Moroccan origin may also be needed to foster informed decision-making.
